# The Impact of Attention on Judgments of Frequency and Duration

**DOI:** 10.1371/journal.pone.0126974

**Published:** 2015-05-22

**Authors:** Isabell Winkler, Madlen Glauer, Tilmann Betsch, Peter Sedlmeier

**Affiliations:** 1 Chemnitz University of Technology, Chemnitz, Germany; 2 University Medical Center of Jena, Jena, Germany; 3 University of Erfurt, Erfurt, Germany; Universidad de Granada, SPAIN

## Abstract

Previous studies that examined human judgments of frequency and duration found an asymmetrical relationship: While frequency judgments were quite accurate and independent of stimulus duration, duration judgments were highly dependent upon stimulus frequency. A potential explanation for these findings is that the asymmetry is moderated by the amount of attention directed to the stimuli. In the current experiment, participants' attention was manipulated in two ways: (a) intrinsically, by varying the type and arousal potential of the stimuli (names, low-arousal and high-arousal pictures), and (b) extrinsically, by varying the physical effort participants expended during the stimulus presentation (by lifting a dumbbell vs. relaxing the arm). Participants processed stimuli with varying presentation frequencies and durations and were subsequently asked to estimate the frequency and duration of each stimulus. Sensitivity to duration increased for pictures in general, especially when processed under physical effort. A large effect of stimulus frequency on duration judgments was obtained for all experimental conditions, but a similar large effect of presentation duration on frequency judgments emerged only in the conditions that could be expected to draw high amounts of attention to the stimuli: when pictures were judged under high physical effort. Almost no difference in the mutual impact of frequency and duration was obtained for low-arousal or high-arousal pictures. The mechanisms underlying the simultaneous processing of frequency and duration are discussed with respect to existing models derived from animal research. Options for the extension of such models to human processing of frequency and duration are suggested.

## Introduction

In everyday life, it is very rare that a given object or event is encountered only once. Usually, one meets people and comes across things (or types of things) repeatedly, and this information about the frequency of occurrence is connected with the information about the length of exposure to these events. Therefore, judgments about these two kinds of magnitudes, one countable (frequency of occurrence) and one uncountable (duration), are likely to be related. Consistent with the majority of research with human participants (see [1], and the contributions therein), we use the term *frequency* to denote the number of times a given stimulus or type of stimulus is presented within a given time interval. Typically, people are remarkably sensitive to the frequency and duration of stimuli [2–10]. However, if these two kinds of judgments depend on a common magnitude system, there could be a mutual biasing effect. Thus, the question arises whether people can always trust in their perception of frequency and duration. There is indeed convincing evidence for such a common magnitude system for numerical and temporal processes [2,11]. On the basis of animal research [12–17] as well as investigations with human subjects [3,18–20], it can be concluded that the same mechanisms are used for the processing of frequency and duration. Moreover, neurophysiological research identified brain areas that are responsible for the processing of frequency as well as duration [21].

If there really exists a common magnitude system that is used for judgments of both frequency *and* duration, both kinds of stimulus information should contribute to the magnitude representation. This, as already mentioned, might yield biased estimates. For example, if a stimulus is presented 10 times, short stimulus presentations might lead to lower frequency estimates compared to longer presentations of the same stimulus (with the same presentation frequency). This reasoning also holds for the other possible kind of mutual impact: If the overall stimulus duration is, say, 10 s, having presented the stimulus 10 times (with a duration of 1 s each) might lead to a higher total duration estimate than having presented it 5 times (with a duration of 2 s each). In sum, the frequency of occurrence of stimuli should have a (biasing!) effect on the judgment of their duration, and vice versa.

Whereas results from animal research conform to the postulated mutual impact of the two kinds of magnitudes, to date, the results of behavioral research with human subjects are largely inconsistent with such an assumption. The usual finding has been an asymmetrical relationship between frequency and duration (e.g., [22–24]), suggesting that duration judgments are influenced by the frequency of occurrence of stimuli. In contrast, frequency judgments seem *not* to be influenced by presentation duration [3,18,23,24].

What might the reason be for this discrepancy between results in animal studies and those in studies with human participants? One difference between the two kinds of research is immediately evident: Animals are usually exposed to stimuli that are very important to them, while the stimuli used in studies with human participants might not rouse the latter’s interest very much. So it is quite likely that the attention directed to experimental stimuli plays a crucial role in these kinds of judgments and is responsible for the differential results. In animal research, the stimuli—usually linked with food items—can be expected to attract a large amount of attention, whereas the stimuli used in research with humans, such as three-letter words on a computer screen, probably do not draw much of participants’ attention. A low level of attention toward experimental stimuli should not have a strong impact on frequency judgments because frequency processing seems to need only minimal attention (e.g., [25,26]). In contrast, adequate duration processing seems to be highly dependent on attentional resources (e.g., [27,28]). Thus, low attention to the stimuli might be responsible for the asymmetrical results in previous studies on the mutual impact of frequency and duration.

In the present study, we explored this by systematically varying the amount of attention in the simultaneous processing of stimulus frequency and duration. Our main question was whether the amount of attention directed to the stimuli has a systematic impact on the extent of bias that one quantity exerts on the other. If this is the case, one should expect the usual asymmetrical results when stimuli receive a low amount of attention. However, when a high amount of attention is directed to the stimuli, the mutual impact of frequency and duration should be more symmetrical. Before we discuss previous results on the mutual impact of frequency and duration, we briefly describe the characteristics of the processing of frequency and duration separately, in more detail.

### Judgments of Frequency

In research on the processing of quantity, different kinds of information have been used. In some cases, digits were presented to the participants (e.g., [20,29,30]), and sometimes a varying number of stimuli shown at the same time were used (e.g., [30–32]), but most often, stimuli were presented successively multiple times (e.g., [3,18, 19, 23,33, 34]). In our experiments we were concerned only with the latter, that is, with the frequency of occurrence, which we refer to simply as frequency.

Sensitivity to event frequencies seems to be a very basic ability. Even young children and animals are able to detect differences in frequency especially for small numbers below 10 [5,12,15,35,36]. Hasher and Zacks [25,26] argued that frequency processing is an automatic encoding process; that is, people are able to make accurate frequency estimates without intentional counting (see also [37]). Frequency judgments therefore should need only minimal attentional resources. Even under difficult circumstances, such as stress, high arousal, or simultaneous processing demands, frequency estimates remain intact and relatively robust against biases [26]. Still, the accuracy of frequency judgments is improved by deeper levels of processing, which are associated with higher amounts of attention being allocated to the stimuli [38–42]. For example, using an orienting task, Johnson et al. [41] manipulated the amount of attention participants directed to stimuli at the encoding stage. The most accurate frequency judgments were obtained in the condition requiring the highest amount of attention to the stimuli. Overall, sensitivity to frequency seems to be a stable characteristic of human perception (for an overview, see [1]), although any factor that affects the amount of attention allocated to the stimuli might be expected to have an effect on the corresponding frequency estimates.

### Judgments of Duration

Humans, like most animals, have a highly developed sensitivity to duration, and duration estimates can be surprisingly accurate (see [6,7]; for an overview of human time perception, see also [43]). Yet, despite an apparent sensitivity to temporal information across a wide range of intervals (from milliseconds to circadian timing; e.g., [44]), the accuracy of duration judgments differs. Sensitivity to very brief durations (below 500 ms) appears to be extremely high and based on processes that are automatic [45]. This kind of duration processing, therefore, needs just low attentional resources [44]. The ability to make duration judgments for periods of seconds or minutes, however, seems to be highly dependent on attentional processes [3,27,28,44]. Thus, Lewis and Miall [46,47] proposed two different time measurement systems (automatic vs. cognitively controlled timing) based on two distinct neural systems. According to this model, the timing of seconds and minutes requires attention, and therefore time perception is a function of the amount of attention directed to the temporal information. Because attentional resources are limited in our cognitive system, temporal and nontemporal information in a task compete for these attentional resources [48,49]. If attention is distracted from time-relevant information, duration judgments are characterized by shorter estimates and lower accuracy [28,50].

A variety of additional factors make duration judgments error prone; some are personal (e.g., a person’s temporary arousal or age; [10,51,52] and others situational (e.g., the complexity of the stimuli; [53]).

### The Mutual Impact of Frequency and Duration Processing

Comparing the results for judgments of frequency and duration, it is evident that the amount of attention directed to the stimuli has an effect on both kinds of judgments, with a higher amount of attention tending to yield more accurate estimates. Yet there is also a pronounced discrepancy in the amount of attention required for the processing of the two types of information: Whereas judgments of frequency seem to work well with low attentional demands, adequate judgments of duration seem to need substantial attentional resources allocated to the stimuli. Despite this discrepancy, as already mentioned, results from animal research (e.g., [14,16,17]) and from research with humans that mimicked the procedures used with animals (e.g., [8]) suggest a common magnitude system for the perception of frequency and duration (e.g., [2,11]; but see [54]).

The most prominent psychological model to explain the simultaneous processing of duration and frequency seems to be the accumulator model, proposed by Meck and Church [16] as a modification of a model previously suggested by Gibbon [55]. In this model that was largely developed in the context of animal research, a pacemaker emits pulses that are summarized by an internal accumulator and eventually stored in memory. A switch controls the accumulation process. It can operate in run-and-stop mode, serving as a timer, or in event mode, serving as a counter. To process the duration of events, pulses are gated to the accumulator for as long as an event is attended to. However, to process the frequency of events, pulses are gated to the accumulator each time an event occurs. The stored values of the accumulation processes will then be compared to existing values of a definite time span or a certain quantity stored in the reference memory. Gallistel and Gelman [56] argued that the accumulation process works for both countable (i.e., frequency) and uncountable (e.g., duration) quantities. Consistent with such an accumulator model, a common neural basis of frequency and duration processing has been identified (the thalamo–cortico–striatal circuits; [11,44,57]. Confirming evidence also comes from a functional magnetic resonance imaging study, in which Dormal et al. [21] found the right intraparietal sulcus to be responsible for the encoding and accumulation stages of Meck and Church’s [16] accumulation model of frequency and duration processing.

Although the existence of common mechanisms for the processing of frequency and duration in the brain implies that these kinds of judgments mutually influence each other, as yet, a substantial amount of evidence with human participants contradicts this assumption. In a pioneering study, Hintzman ([23], Experiment 3) investigated the mutual influence of frequency and duration processing by varying both stimulus frequency and presentation duration for three-letter words. Frequency and duration estimates revealed an asymmetrical result pattern: Frequency judgments were quite accurate and only marginally influenced by the presentation duration of the stimuli. Duration judgments, however, were not remotely as accurate and were strongly affected by stimulus frequency. This asymmetrical effect was replicated in later studies [22,24,34].

Roitman et al. [19] also obtained an asymmetrical relationship between judgments of frequency and duration. Using a numerical and a temporal bisection task, they examined people’s ability to discriminate stimuli (flashes) by either frequency or duration. Consistent with previous research, participants’ sensitivity to frequency was higher than their sensitivity to duration; however, when participants were asked to focus their attention on either stimulus frequency or stimulus duration, sensitivity to the respective magnitude increased. Droit-Volet et al. [18] used a similar task to investigate the ability of adults and children to discriminate stimuli in respect to frequency and duration. They found that frequency strongly interfered in a temporal bisection task, whereas participants’ performance in a numerical bisection task was uninfluenced by stimulus duration. In another experiment, Dormal et al. [3] likewise examined participants’ ability to distinguish the frequency and duration of flashing dots by means of a Stroop-task paradigm and obtained the often-found asymmetry between judgments of frequency and duration. The frequency discrimination task was performed with little error even with incongruent stimulus pairs (i.e., high frequency linked to short presentation duration and vice versa). The duration discrimination task, in contrast, was characterized by a higher error rate within the incongruent stimulus condition. Thus, stimulus frequency had an effect on duration processing, but frequency processing was barely influenced by presentation duration.

The asymmetrical relationship found in these studies makes perfect sense when considering what is known about the attentional demands of frequency and duration processing. Frequency processing, as already mentioned, is a relatively automatic process and therefore requires only minimal attentional resources. Judgments should be comparatively reliable (i.e., not or not strongly biased by the presentation duration) even when the stimuli are not the focus of participants’ attention. Duration processing, on the other hand, needs more attentional resources. If the participants hardly focus their attention on the stimuli or if attentional resources are exhausted by a high cognitive load for the task (e.g., the requirement of a secondary task), duration judgments should be less accurate and—if duration is processed together with stimulus frequency—biased by the more stable magnitude: frequency.

Winkler and Sedlmeier [58] demonstrated that cognitive load indeed has a strong impact on frequency and duration judgments. In conditions that require a large number of stimuli to be processed simultaneously, a more asymmetrical relationship between frequency and duration was found compared to conditions with a lower number of different stimuli. Furthermore, the performance of a secondary task in addition to the processing of frequency and duration resulted in lower sensitivities to frequency and duration, as well as a lower mutual influence of frequency and duration compared to a condition without a secondary task (see also [59], for the strong impact of a secondary task on duration judgments).

Although a large number of studies exist that obtained an asymmetrical relationship between frequency and duration, Javadi and Aichelburg [31] found in a study with human participants a mutual impact of numerical and temporal information. However, this study did not present stimuli successively but instead manipulated the numerical information by varying the number of simultaneously presented stimuli (dots) on a computer screen. Moreover, the stimulus durations were in the millisecond range, a timescale that seems to depend on processes other than those used in interval timing [44]. Nonetheless, it might eventually turn out that there exists a magnitude system that covers different aspects of numerical judgments and different timescales.

In the present experiment, we systematically varied the amount of attention allocated to the stimuli. We directed the participants’ attention to the stimuli in an intrinsic and an extrinsic way. To manipulate attention intrinsically, we varied the type of stimulus (names vs. pictures) as well as the arousal potential of the stimuli (low- vs. high-arousal stimuli) resulting in three stimulus conditions: (a) names, (b) low-arousal pictures, and (c) high- arousal pictures. Because pictures generally provide more visual detail compared to words, this stimulus type can be expected to absorb more attention (e.g., [60]). We also expected high-arousal pictures to attract more attention compared to names and low- arousal pictures, because high-arousal stimuli are typically rated as more interesting compared to low-arousal stimuli, and the mean voluntary exposure time is longer for high- arousal stimuli [61]. To manipulate attention extrinsically, we varied a physical effort requirement during the stimulus presentation. Participants had to lift a moderately heavy dumbbell when—and for as long as—they saw a certain stimulus category and were asked to just rest the arm when they saw another stimulus category. To comply with the experimental demands, in the physical effort condition participants had to attend to the complete stimulus presentations to keep the dumbbell lifted for as long as the relevant stimulus was shown on the screen and, therefore, had to direct a higher amount of attention to the stimuli.

We assumed that both the kind of stimuli and physical effort would moderate the perception of stimulus frequency and duration. Thus, we expected that sensitivity to frequency and sensitivity to duration would be higher when more attention was directed to the stimuli in either an intrinsic or an extrinsic way. We expected to see the strongest effects for high-arousal pictures processed in the physical exercise condition and the smallest effects for names with participants resting their arms. Furthermore, we hypothesized we would find an asymmetrical relationship between frequency and duration when less attention was directed to the stimuli: that is, a large influence of stimulus frequency on duration judgments, and only a low effect of presentation duration on frequency judgments. In contrast, in conditions with higher amounts of attention to the stimuli, we assumed we would find a more symmetrical mutual influence between frequency and duration.

## Method

### Participants

Seventy-five undergraduates (63 female, 12 male, *M*_age_ = 22 years, *SD* = 5.5) at the Chemnitz University of Technology participated in the experiment and received course credit for their participation.

### Ethical Approval

The investigation was conducted according to the principles expressed in the Declaration of Helsinki and in accordance with relevant institutional and national guidelines and regulations, that is, the guidelines of the German Psychological Society [62] and the regulations of the Chemnitz University of Technology [63]. We strictly adhered to all criteria mentioned there. A formal approval of the study by the Ethics Committee of Chemnitz University of Technology was not mandatory, since the study adhered to all the required regulations.

All participants gave their oral informed consent in accordance with the guidelines of the Ethics Committee of Chemnitz University of Technology. The informed consent was oral because the data were analyzed anonymously. Participants were ensured strict confidentiality before participating in the study. They were then informed about the objectives and the procedure of the investigation as well as about their right to withdraw from the study at any time without adducing reasons and without any negative consequences. Consent of the participants was documented by means of the examiner’s signature on the protocol sheet of the investigation. The experiment did not start without the approval of the participant that she or he understood the examiner’s information in detail.

### Experimental Design

To manipulate the kind of stimuli (between subjects) to attract various amounts of attention, participants were presented with either first names, low-arousal pictures, or high- arousal pictures. The manipulation of the second independent variable, physical effort, consisted in having participants (within subjects) either lift a dumbbell (high physical effort) or rest their arm (low physical effort) while a certain stimulus was displayed. Stimulus frequency was varied (within subjects) by presenting each stimulus two, four, or eight times. Stimulus duration was varied (also within subjects) by modifying the total presentation duration of a stimulus to be 16, 24, or 32 s. Hence, for example, if a stimulus was in the four times/24 s condition, it was presented four times for 6 s each time.

### Material

The three kinds of stimuli presented to the participants were: (a) 18 different first names, (b) 18 pictures with low-arousal content, and (c) 18 pictures with high-arousal content. The first names were selected from a study by Rudolph, Böhm, and Lummer [64] that provided word norms for a large sample of German first names. From this study, we selected 36 names (18 female and 18 male names, see S1 Table) with a similar length (two to three syllables) that scored within the second and third quartile in popularity and were perceived as being relatively modern. Each participant in the names condition was presented a random sample of 18 first names (9 female and 9 male). Furthermore, we selected 18 low- and 18 high-arousal pictures from the International Affective Picture System (IAPS; [65]). The IAPS is a collection of pictures that have been evaluated on their power to evoke emotions, rated on scales of 1 (*low*) to 9 (*high*) for emotional valence as well as for intensity of arousal. We selected pictures that evoked only positive emotions (valence ratings over 4.8) to avoid frightening the participants or causing disgust. Also, for negative emotions participants’ reactions are less predictable. Phobic or disgusting stimuli could either attract more attention due to an alertness reaction or be avoided. Studies on the effect of stimulus emotionality on the allocation of attention showed that the arousal level, not the valence of the stimuli, influences the amount of attention participants direct to the stimuli [61,66]. Therefore, we selected pictures with either low emotional arousal (arousal ratings below 3.0) or high emotional arousal (arousal ratings over 5.5) to vary the arousal potential of the pictures, and thus assumably, the amount of attention directed to the stimuli. For the low-arousal pictures the mean arousal rating was 2.76 (*SD* = 0.17) and the mean valence rating was 5.62 (*SD* = 0.65); for the high-arousal pictures the mean arousal rating was 6.34 (*SD* = 0.55) and the mean valence rating was 6.87 (*SD* = 0.92). For both picture types, we selected 9 pictures illustrating humans (engaged, for example, in reading or playing chess for low emotional arousal, and in extreme sports or erotic actions for high emotional arousal) and 9 pictures illustrating objects (such as a plant or a boat for low emotional arousal, and lightning or a rocket for high emotional arousal). The presentation of the stimuli and the assessment of the dependent measures were controlled by a computer program (E-Prime 2.0 software; [67]) run under a Windows XP environment with 17-inch monitors.

### Procedure

Participants were randomly assigned to one of the three between-subjects conditions (names, low-arousal pictures, and high-arousal pictures). All participants were tested in separate cubicles. Upon arrival, they were informed that they were to watch a series of stimuli (either names or pictures) appearing consecutively on the computer screen and that they would be expected to answer several questions about these stimuli after the complete stimulus presentation. Of the 18 stimuli of each type, 6 were presented two times, 6 were presented four times, and 6 were presented eight times. Thus, every participant saw 84 successive stimulus presentations (in an individually randomized order) with the restriction that a given stimulus was not shown twice in succession. The inter-stimulus interval was always 2 s. The participants were not explicitly informed that they were to estimate stimulus frequency and duration in order to avoid deliberate counting strategies. However, participants were asked to attend to the stimulus presentations, and they were briefed that stimuli could occur repeatedly and with varying presentation durations.

Participants’ physical effort during the stimulus presentation was varied by having them either (a) lift a moderately heavy dumbbell (5 pounds for women, 10 pounds for men) with their nondominant arm each time—and for as long as—they saw a stimulus of a certain category or (b) just rest the arm when they saw a stimulus of another category. Within the names condition, the two stimulus categories were female and male names. Within the pictures conditions the categories were pictures showing humans and objects. Exemplars of the stimulus categories in each between-subjects condition appeared in a randomly mixed order. The assignment of the categories to the physical-effort conditions was counterbalanced across participants. To foster participants’ commitment to the appropriate accomplishment of the exercise task, electromyogram electrodes were attached to the biceps of the participants’ nondominant arm and they were told that the muscle tension would be recorded. However, actual measurement of the muscle activation did not take place.

During a training phase that lasted about 2 min, participants were familiarized with the presentation format of the stimuli as well as with the dumbbell exercise. Only stimuli that would not be included in the subsequent experimental trials were presented.

After the presentation of stimuli, participants were required to estimate the presentation frequency and the total presentation duration of each of the 18 presented stimuli. Therefore, the stimuli were presented two more times, each time in a newly randomized order. In the two trials, the stimuli appeared one after another on the screen with the instruction to estimate (a) the presentation frequency of each stimulus and (b) the total presentation duration of each stimulus. One half of the participants were asked first to estimate the stimulus frequency and then to estimate the respective stimulus durations. The order was reversed for the other half of the participants. For both frequency and duration judgments, participants were explicitly informed about the lowest and highest possible values (i.e., two to eight presentations for the frequency judgments, and 16 to 32 s for the duration judgments) in order to prevent extreme judgments. The experiment took about 30 min. Participants were debriefed at the end of the experiment.

### Statistical Analysis

Note that our design included experimental variation both within (stimulus frequency, presentation duration, and physical effort) and between participants (kind of stimulus). Analyzing such a design, that is, variables from two different levels at one single common level (as is the case for traditional regression analysis) suffers from both statistical and conceptual problems: error estimates can be expected to be wrong and one could arrive at unjustified conclusions [68,69,70].

One might consider mixed-design analyses of variance as an alternative but these suffer from the problem that they are only suited to examine “omnibus hypotheses” and not precise research hypotheses (see [71,72]). In our case, we had exact hypotheses for both within- and between-subjects factors. The best (and also most elegant) way to examine the effects in a mixed design that we are aware of is multilevel regression analyses using hierarchical linear models (see [68,73]. The following analyses were performed with such a model using HLM software [74]. For each analysis, the variations in stimulus frequency and presentation duration, respectively, are modeled at level 1, nested in the number of participants included in the respective analysis modeled at level 2 (see S1 Data File). An additional advantage of multilevel regression analyses over mixed-design analyses of variance in the current study is that they can explain how the manipulations of attention affect the interaction of frequency and duration.

In what follows, we report the results of the data analyses in two steps. First, we report the four main effects of frequency and duration across all attention conditions: (a) the extent to which participants were sensitive to stimulus frequency (i.e., the effect of stimulus frequency on frequency judgments), (b) the participants’ sensitivity to total presentation duration (i.e., the effect of stimulus duration on duration judgments), (c) the effect of stimulus frequency on duration judgments, and (d) the effect of stimulus duration on frequency judgments. Also, we examine the influence of attention on the size of these four main effects of frequency and duration, that is, the impact of the intrinsic (stimulus type) and extrinsic attention manipulation (physical effort) on the sensitivities to frequency and duration and their mutual influence. Second, we report the size of frequency and duration effects separately for the specific attention conditions to make the interdependency between the two magnitudes in the particular attention conditions more salient for comparison.

## Results

### Main effects of frequency and duration and the impact of attention

To calculate the participants’ sensitivity to frequency, we used frequency judgments as dependent variable and the actual levels of stimulus frequency as predictor. For the calculation of the duration effect on frequency judgments, we again utilized frequency judgments as dependent variable and the actual stimulus duration as predictor. The same principle was applied for the calculation of the sensitivity to duration and the effect of frequency on duration judgments. For each multilevel regression analysis, we always controlled for the nonfocal-magnitude level (stimulus frequency and presentation duration, respectively) by entering this variable into the analyses as additional predictor. To examine the statistical significance of the coefficients, the regression coefficients were tested against zero by means of a one-tailed one-sample *t* test. For each of the main effects of frequency and duration, we tested in only one relevant direction: The higher the level of stimulus frequency/stimulus duration, the higher the frequency judgments/duration judgments.

Table 1 displays the results of the multilevel regression analyses across all attention conditions. The top part of the table contains the four main effects of frequency and duration (represented in multilevel analyses as variations in the intercept), illustrating that the sensitivities to frequency and to duration are generally reliable. Although the effects are not symmetrical in size, frequency and duration influence each other mutually, demonstrated by a *p* value below. 001 for the effect of frequency on duration judgments and a somewhat higher *p* value for the effect of duration on frequency judgments.

**Table 1:** Coefficients from the two-level regression model predicting the four main effects of frequency and duration (intercept) and the interactions of these effects with stimulus type and physical effort (slopes).

Effects	Regression coefficient	Standard error	*t*	*p*
*Intercept (main effects)*				
Sensitivity to frequency	0.33	0.01	25.2	<.001
Sensitivity to duration	1.36	0.17	7.86	<.001
Frequency effect on duration	0.55	0.04	13.0	<.001
Duration effect on frequency	0.18	0.04	4.31	<.001
*Slope (stimulus type)*				
Sensitivity to frequency	0.06	0.02	3.53	<.001
Sensitivity to duration	0.91	0.19	4.77	<.001
Frequency effect on duration	0.11	0.05	2.10	.018
Duration effect on frequency	0.12	0.05	2.44	.008
*Slope (physical effort)*				
Sensitivity to frequency	0.02	0.01	1.82	.035
Sensitivity to duration	0.39	0.16	2.34	.010
Frequency effect on duration	0.04	0.03	1.19	.117
Duration effect on frequency	0.10	0.04	2.62	.005

*Note*. The *p* values are one tailed; *df* = 1.269 in each case.

However, the focus of the overall multilevel regression analyses is the interaction effect of the attention manipulations on the four main effects of frequency and duration. That is, we tried to answer the question of how strong the impact of attention was on the sensitivities to frequency and duration as well as on their mutual influence. Therefore, we entered stimulus type (names, low- arousal and high-arousal pictures) and physical effort (low vs. high effort) as additional predictors into the analyses. To calculate the interaction effect of stimulus type, we contrast coded this variable (i.e., names = -1; low-arousal pictures = 0; high-arousal pictures = 1) and entered it into the analyses as an interaction term of stimulus type and the respective magnitude level (stimulus frequency and presentation duration) of the stimuli. For example, to calculate the effect of stimulus type on the sensitivity to frequency, we used the frequency judgments as dependent variable and the product of (contrast-coded) stimulus type and level of (centered) stimulus frequency as predictor. The same procedure was applied to calculate the effect of physical effort (contrast coded as low effort = -1; high effort = 1) on the four main effects of frequency and duration.

The second part of Table 1 displays the interaction effect (represented in multilevel analyses as slope) of the first attention variable, *stimulus type*, on the four main effects of frequency and duration. The results demonstrate that stimulus type has a large effect on sensitivity to frequency and to duration; that is, the more attention the stimuli were expected to attract, the better were the participants in discriminating between the three levels of frequency and duration. Stimulus type also reliably affected the mutual influence between frequency and duration. Again, the more attention the stimuli were assumed to attract, the stronger the mutual influence between frequency and duration.

The results for the extrinsic attention manipulation, *physical effort*, are displayed in the third part of Table 1. Physical effort had a systematic effect on sensitivities to frequency and duration: When stimuli were processed under physical effort, participants were more sensitive to differences in the levels of frequency and duration. Whereas physical effort had no significant effect on the frequency effect on duration, we obtained a reliable effect of physical effort on the duration effect on frequency.

One might argue that there is a potential problem with multiple comparisons. Whereas some authors claim that alpha-adjustments are not needed at all [75], a common recommendation suggests to perform such adjustments only to focal tests [76]. If we apply this recommendation to our case, this refers to the tests about the impact of duration on frequency judgments. The three respective tests in Table 1 still exhibit a level that is lower than the cutoff one would arrive at using the rather conservative Bonferroni correction, based on an overall alpha of. 05 (alpha_adjusted_ = .017).

In what follows, we report the size of the four main effects of frequency and duration separately for the different attention conditions and graphically illustrate the variations in the judgments of frequency and duration over the experimental conditions to facilitate the evaluation of the impact of attention on the relationship between frequency and duration.

### Main effects of frequency and duration separately for the different stimulus types processed under low and high physical effort

**Fig 1:**
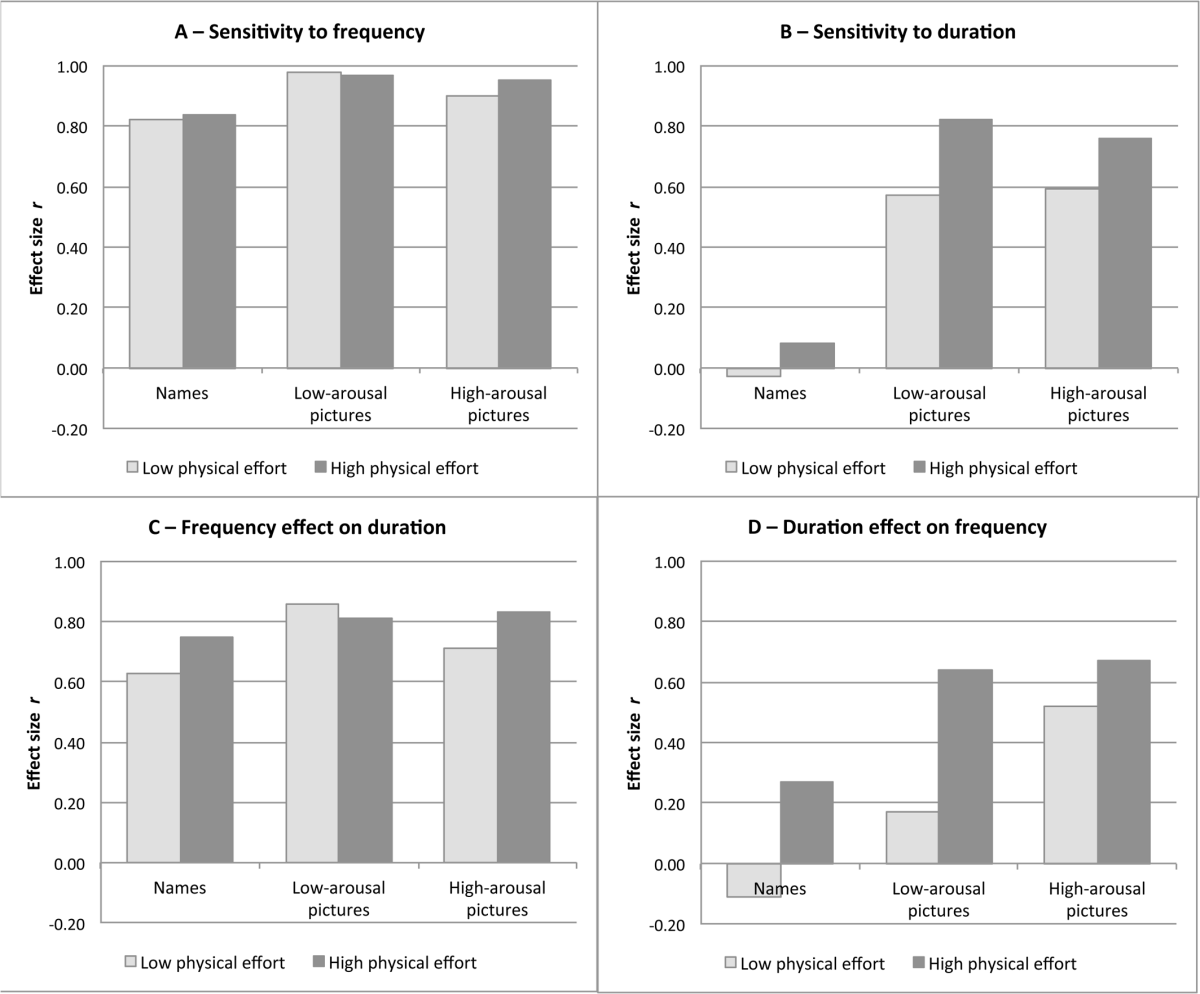
Effect size *r* of the regression coefficients in the multilevel regression analyses separately for the different stimulus types processed under low and high physical effort. A) Sensitivity to stimulus frequency; B) Sensitivity to stimulus duration; C) Influence of stimulus frequency on duration judgments; D) Influence of stimulus duration on frequency judgments. The figure shows generally large effect sizes for the sensitivity to frequency and for the frequency effect on duration in every attention condition. However, sensitivity to duration was obtained only in the picture conditions with especially large effects when pictures had to be processed under high physical effort. The duration effect on frequency was low for names in both effort conditions and for low-arousal pictures processed under low effort (and somewhat larger for high-arousal pictures under low effort). For both picture types processed under high effort a large duration effect on frequency was obtained.

The results of the separate multilevel regression analyses for each of the six attention conditions (names, low-arousal and high-arousal pictures processed under low vs. high physical effort) are summarized in Table 2. To facilitate the comparison of the main effects of frequency and duration between the attention conditions, we calculated the effect size *r* from *t* values [72,77], for each regression coefficient, and plotted these effects against each other (see Fig 1A–1D). Means and standard deviations of the judgments of frequency and duration for each of these experimental conditions are displayed in S2 Table. In the following, we discuss the judgments of frequency and duration as well as their mutual influence in detail.

**Table 2:** Coefficients from the two-level regression model predicting the main effects of frequency and duration for names, low- and high-arousal pictures processed under low and high effort.

Effects	Regression coefficient	Standard error	*t*	*p*
*Names/low effort*				
Sensitivity to frequency	0.22	0.03	7.07	<.001
Sensitivity to duration	-0.05	0.31	-0.15	.440
Frequency effect on duration	0.35	0.09	3.94	<.001
Duration effect on frequency	-0.07	0.12	-0.54	.296
*Names/high effort*				
Sensitivity to frequency	0.25	0.03	7.53	<.001
Sensitivity to duration	0.17	0.41	0.41	.345
Frequency effect on duration	0.42	0.07	5.58	<.001
Duration effect on frequency	0.14	0.10	1.38	.090
*Low-arousal pictures/low effort*				
Sensitivity to frequency	0.40	0.02	22.4	<.001
Sensitivity to duration	1.49	0.43	3.38	.001
Frequency effect on duration	0.70	0.08	8.27	<.001
Duration effect on frequency	0.09	0.11	0.83	.208
*Low-arousal pictures/high effort*				
Sensitivity to frequency	0.41	0.02	18.6	<.001
Sensitivity to duration	2.76	0.40	6.93	<.001
Frequency effect on duration	0.68	0.10	6.67	<.001
Duration effect on frequency	0.34	0.08	4.07	<.001
*High-arousal pictures/low effort*				
Sensitivity to frequency	0.31	0.03	9.90	<.001
Sensitivity to duration	1.45	0.41	3.54	.001
Frequency effect on duration	0.49	0.10	5.00	<.001
Duration effect on frequency	0.21	0.07	2.98	.003
*High-arousal pictures /high effort*				
Sensitivity to frequency	0.39	0.03	14.7	<.001
Sensitivity to duration	2.30	0.40	5.81	<.001
Frequency effect on duration	0.69	0.09	7.33	<.001
Duration effect on frequency	0.35	0.08	4.44	<.001

*Note*. The *p* values are one tailed; *df* = 24 in each case.

#### Sensitivity to stimulus frequency

**Fig 2:**
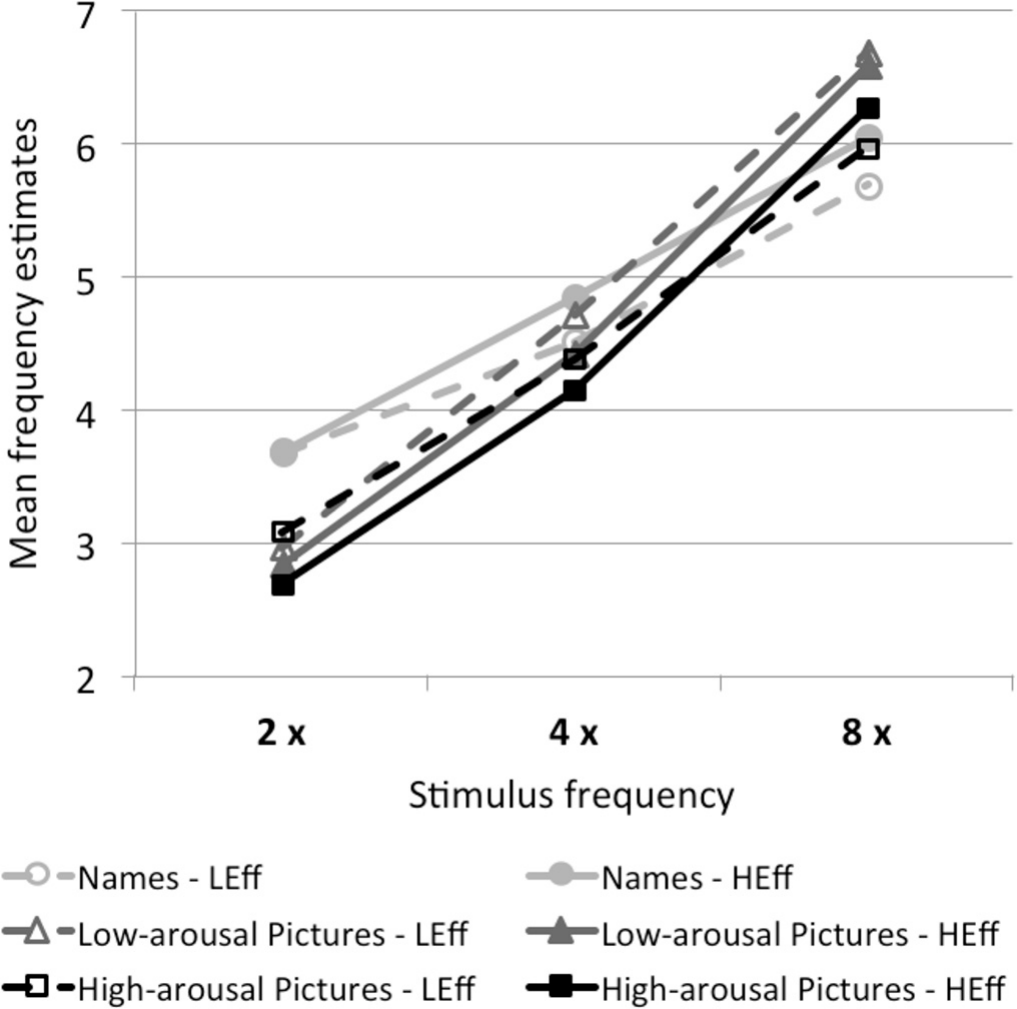
Sensitivity to frequency. Sensitivity to stimulus frequency in the different stimulus-type and physical-effort conditions. Stimuli were presented two (2 x), four (4 x), or eight (8 x) times. Frequency estimates are averaged per participant over the three levels of presentation duration. LEff = low physical effort; HEff = high physical effort. The figure shows a high sensitivity to frequency in each attention condition.

Fig 2 shows the mean frequency estimates for each level of actual stimulus frequency, averaged across the three duration levels. Each of the six lines displays one of the six combinations of stimulus type and physical effort condition. The figure shows that frequency judgments systematically covary with actual stimulus frequency within each of these six attention conditions, resulting in monotonously increasing frequency judgments with increasing stimulus frequency. The results of the multilevel regression analyses support these impressions (see Table 2); that is, the participants were highly sensitive to stimulus frequency in all attention conditions. Fig 1A displays large effect sizes of the regression coefficients in every attention condition.

Despite the participants’ high sensitivity to frequency, the data reveal that low frequencies (two repetitions) were generally overestimated (*M* = 3.2), whereas high frequencies (eight repetitions) were underestimated (*M* = 6.2). The best frequency judgments were obtained for the condition providing four repetitions (*M* = 4.5). This tendency toward the mean is in line with previous findings on frequency processing (e.g., [33,78,79]).

#### Sensitivity to stimulus duration

**Fig 3:**
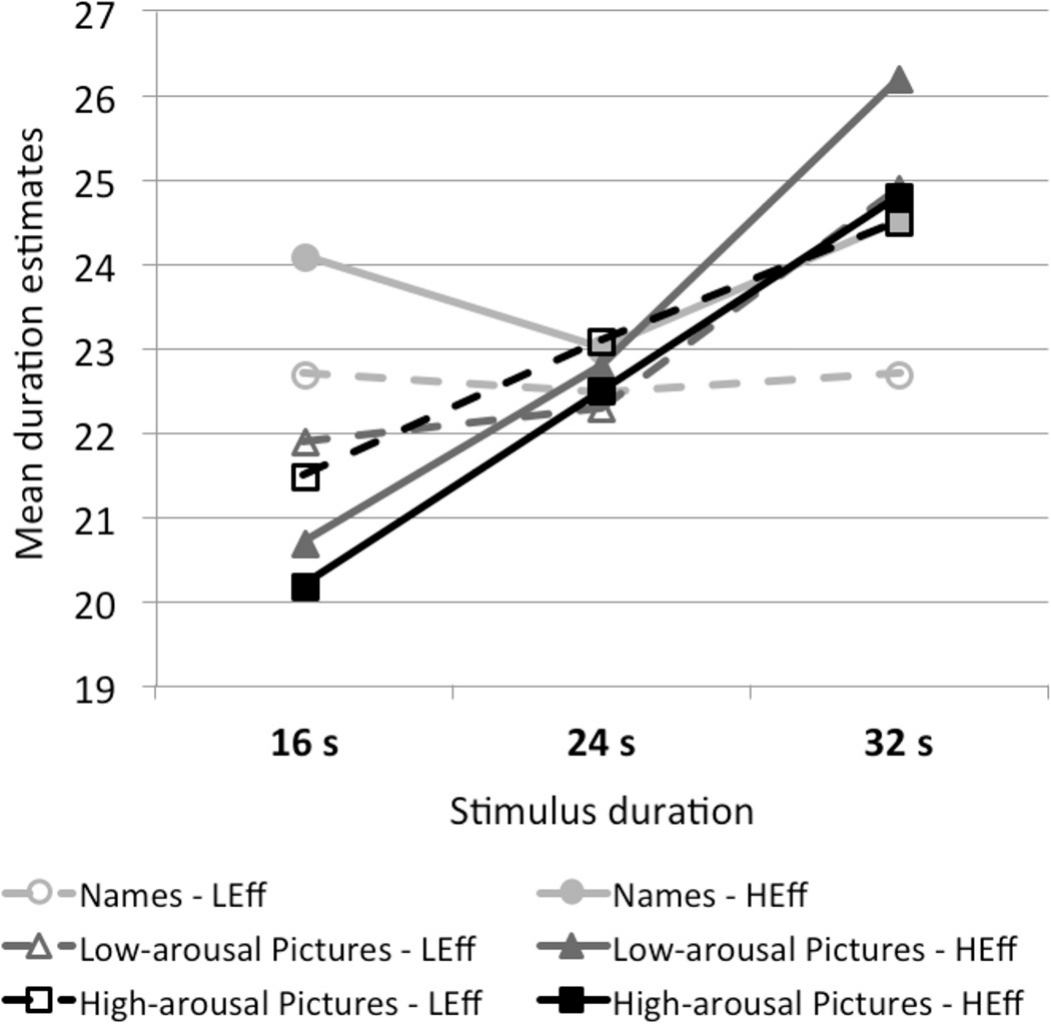
Sensitivity to duration. Sensitivity to stimulus duration in the different stimulus-type and physical-effort conditions. Stimuli were presented for 16 s, 24 s, or 32 s. Duration estimates are averaged per participant over the three levels of presentation frequency. LEff = low physical effort; HEff = high physical effort. The figure displays differences in the sensitivity to duration depending on the stimulus type and physical effort.

Fig 3 shows the mean estimates for total presentation duration, averaged across the three frequency levels. Sensitivity to stimulus duration obviously differs across the attention conditions. The multilevel regression analyses reveal that the sensitivity to duration is reliable in both picture conditions (see Table 2). In the names conditions, however, participants were not sensitive to differences in stimulus duration. Fig 1B displays large effect sizes of sensitivity to stimulus duration only in the picture conditions (no matter if the pictures had a low or high arousal potential). Sensitivity to duration was even higher when pictures were processed under physical effort.

Similar to the frequency judgments, the judgments of total duration reveal a tendency to the mean, that is, short total durations (16 s) were generally overestimated (*M* = 21.9), whereas long durations (32 s) were underestimated (*M* = 24.6). The best duration judgments were obtained for the medial duration condition of 24 s (*M* = 22.7). This effect also occurs when we take only the pictures conditions with a markedly higher sensitivity to duration into account. This “central-tendency” effect (also called Vierordt’s law) is consistent with previous findings on duration processing (see [80]).

#### Effect of stimulus frequency on duration judgments

**Fig 4:**
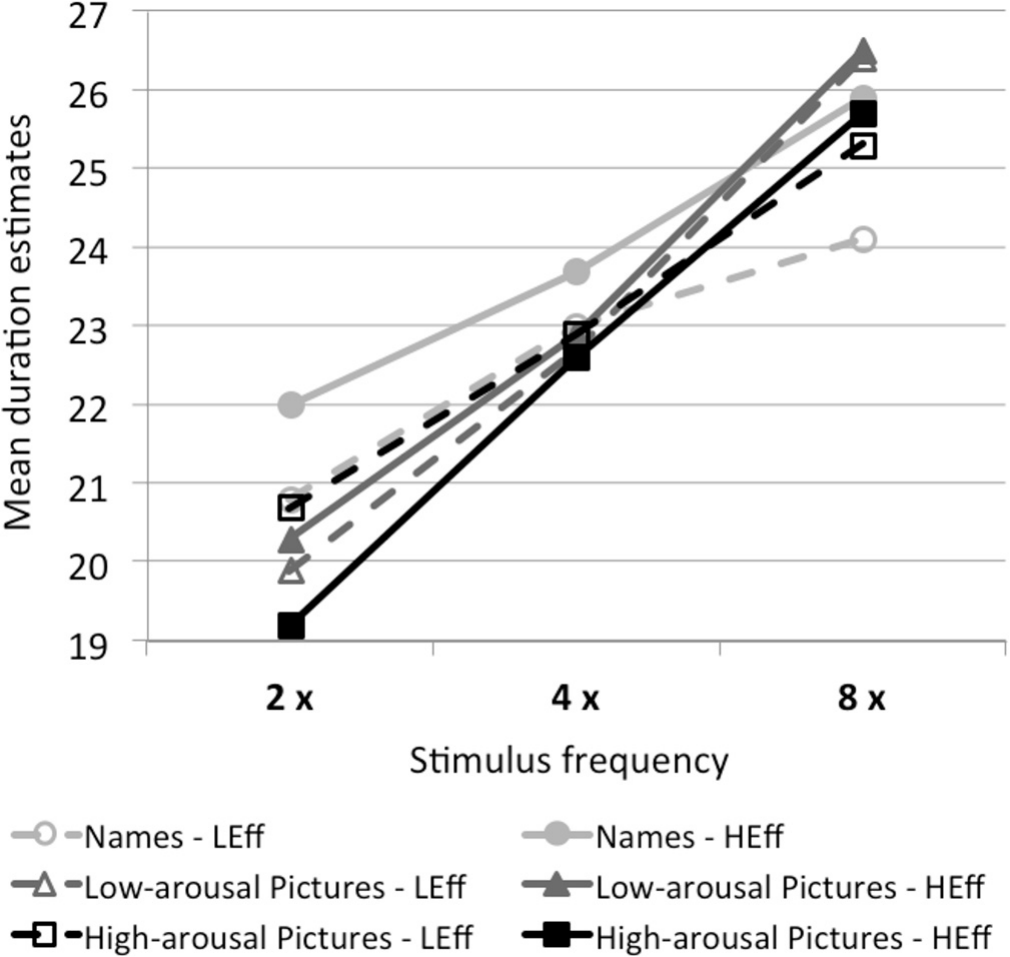
Influence of frequency on duration. Influence of stimulus frequency on duration judgments in the different stimulus-type and physical-effort conditions. Stimuli were presented two (2 x), four (4 x), or eight (8 x) times. Duration estimates are averaged per participant over the three levels of presentation duration. LEff = low physical effort; HEff = high physical effort. The figure displays a large frequency effect on duration in each attention condition.

To better visualize the effect of stimulus frequency on duration judgments, we used estimates for total presentation duration averaged across the three duration levels (see Fig 4). Within each attention condition, we obtained a high covariation between stimulus frequency and duration estimates. The results of the multilevel regression analyses reveal a reliable frequency effect on duration estimates (see Table 2). Large effect sizes of the regression coefficients are obtained in each of the four attention conditions (Fig 1C).

#### Effect of stimulus duration on frequency judgments

**Fig 5:**
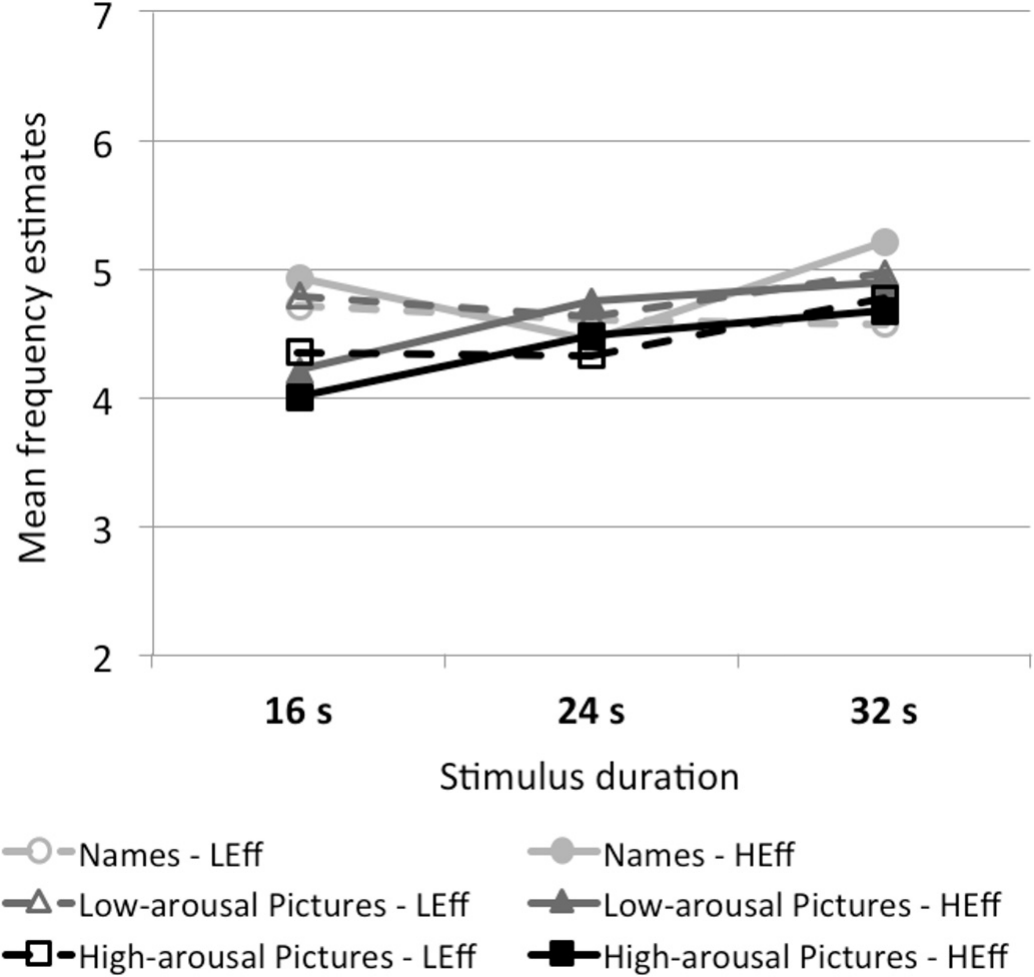
Influence of duration on frequency. Influence of stimulus duration on frequency judgments in the different stimulus-type and physical-effort conditions. Stimuli were presented for 16 s, 24 s, or 32 s. Frequency estimates are averaged per participant over the three levels of presentation frequency. LEff = low physical effort; HEff = high physical effort. The figure shows a systematic covariation of the frequency judgments with presentation duration only in the conditions in which pictures were processed under high effort.

A summary of the differences in covariation of duration judgments with stimulus frequency for the four attention conditions is shown in Fig 5. Frequency judgments only slightly covary with total presentation duration in some of the attention conditions. For names, the multilevel regression analyses reveal no significant duration effects on frequency judgments (see Table 2). Also for low-arousal pictures processed under low effort, the analysis yields no significant effect. However, for low-arousal pictures processed under high effort and for high-arousal pictures processed under low and high effort, a significant duration effect was obtained. Fig 1D shows the differences in the duration effect on frequency in terms of effect size. Only when the two attention factors were combined (pictures processed under high physical effort), a large duration effect on frequency judgments emerged.

### Summary of the Results

The results are consistent with our expectation that the amount of attention directed to the stimuli would have an impact on the sensitivities to frequency and duration as well as on the degree of the mutual influence of frequency and duration. Apart from a generally high sensitivity to frequency, we found a markedly higher sensitivity to duration for pictures (with low and high arousal potential) compared to names. Sensitivity to duration was additionally increased by high physical effort. An effect of frequency on duration (similar to sensitivity to frequency) was obtained in each experimental condition and increased when pictures were presented instead of names. However, physical effort did not cause a systematic additional increase in this effect. An effect of duration on frequency was not obtained in the condition with the weakest attention manipulation: names processed under low physical effort. Only for pictures processed under high physical effort did we find a large duration effect on frequency judgments and, hence, a large mutual influence between frequency and duration. In the medial attention conditions—the names condition with high physical effort and the pictures conditions with low physical effort—we obtained only small to moderate duration effects on frequency. Thus, it is only when both attention manipulations were combined that we obtained a high mutual influence between frequency and duration.

Our variations in the arousal potential of the pictures did not yield big differences in the main effects of frequency and duration. It could be that the two stimulus groups did not sufficiently differ in the degree to which they attracted the participants’ attention. Note that we exclusively chose pictures that received positive emotional valence ratings in order not to stress the participants by exposure to violent or disgusting stimulus material. Therefore, we could not vary the arousal level of the stimuli to the maximum.

## Discussion

The general purpose of the study reported here was to test the hypothesis that there is a common mechanism that determines the processing of both frequency and duration, as suggested by neuropsychological research (e.g., [11,21,57]). If this basic assumption holds, the two kinds of information should have a biasing effect on the judgments about the respective other quantity, that is, the frequency of events should (wrongly) influence duration judgments and vice versa. To date, behavioral studies with human participants have not yielded consistent results. In studies that simultaneously varied stimulus frequency and duration, typically an asymmetrical relationship between the two variables was obtained: Frequency judgments were quite accurate and remained basically uninfluenced by stimulus duration. Duration judgments, in contrast, were typically less accurate and were strongly influenced by stimulus frequency.

The results of the present research reveal that the amount of attention directed to the stimuli during encoding plays a crucial role in the processing of frequency and duration. If sufficient attention was focused on the stimuli, participants were sensitive to both stimulus frequency and presentation duration. This was evident as the sensitivity to frequency as well as to duration was significantly higher for pictures than for names. Additionally, sensitivity to frequency and to duration was higher when the stimuli had to be processed under high physical effort requirements. However, the most interesting result is that the asymmetrical relationship between frequency and duration became more symmetrical with an increasing amount of attention directed to the stimuli. The strongest mutual influence of frequency and duration occurred when the two attention manipulations—intrinsic and extrinsic—were combined. In sum, the results are consistent with the assumption that the higher the attention directed to the stimuli, the higher the biasing impact of stimulus duration on stimulus frequency.

As already mentioned, frequency estimation is a relatively automatic process that requires only minimal attentional resources [26]. Therefore, people are quite sensitive to variations in stimulus frequency, even when perceiving a large number of stimuli or when processing personally irrelevant stimuli. The attentional demands of duration estimation, however, are higher. When presented with an additional task, attentional resources become scarce, and consequently, sensitivity to duration decreases [58]. In addition, sensitivity to duration is enhanced when stimuli are relevant to the individual and therefore attract more attention, or when attention is extrinsically directed to the stimuli. It is presumably only in this case that people tend to focus their attention on the stimuli for as long as they are presented; as a consequence, differences in presentation duration are perceived more accurately. Only when the perception of both frequency and duration is relatively accurate do the judgments influence each other.

In the literature on time perception typically a differentiation between prospective and retrospective time estimates is made [81]. The difference is whether the participants paid attention to the duration of an experienced time interval or not. This means that to meet the conditions of prospective time perception, the participants have to pay attention not only to the stimuli themselves, but also to their duration, that is, to the temporal aspect of the stimulus presentation. In experimental research a prospective paradigm is normally created by informing the participants in advance that they will have to estimate the duration of a certain time interval, while in a retrospective paradigm they will be asked for time estimates unexpectedly after the experience of the interval in question. In the current study the participants were not explicitly informed that they would have to estimate stimulus durations. Therefore, the experimental design can be considered retrospective. However, the participants were asked to direct their attention to the stimuli and were informed that the stimuli would differ in terms of presentation duration. Thus, we do not know for sure whether the participants paid attention to the stimulus duration. According to the attentional gate model [10], prospective time estimates depend strongly on the amount of attention directed to the duration of an interval or a stimulus presentation. If the participants do not focus their attention on the duration of an interval (or a stimulus presentation), they have to generate the duration judgment in retrospect on the basis of retrievable memory content for the time interval in question. For both time perspectives, the amount of attention paid to the stimuli plays an important role: In the case of prospective time perception, time estimates are expected to be shorter when the amount of attention directed to the stimuli is high, and hence, the attention is distracted away from the temporal aspects of the stimuli [52]. In the case of retrospective time perception, time estimates are expected to be longer when the amount of attention directed to the stimuli is high, and therefore, the stimulus presentations can be retrieved with a higher probability. However, in the current study, the direction of deviation between the duration judgments and the actual presentation duration (overestimation or underestimation of stimulus duration) was not important. More important was the degree to which the participants were sensitive to variations in stimulus duration as well as to the interdependency between judgments of stimulus duration and stimulus frequency.

Taken together, the present findings strongly argue for the existence of a common mechanism underlying the processing of frequency and duration. We conclude that stimulus duration is processed more deeply the more attention is directed to the stimuli, and it is only when sufficiently high attention is directed to stimuli that a mutual interdependence of frequency and duration appears.

These findings have two implications: First, to accurately estimate the duration of an event—in contrast to frequency estimation—it is necessary to strongly focus on that event. However, in everyday situations with simultaneous processing demands, such as maneuvering in traffic, our duration judgments might be comparatively less accurate. Second, because of common processing mechanisms, our duration judgments might be biased by the frequency of events occurring in a given time span, for example, the frequency of red traffic lights on the way to work. The biases will in principle work in both directions, with stimulus frequency influencing duration judgments and presentation duration affecting frequency estimates, but the latter is less likely to occur.

The development of an explanatory model that integrates both frequency and duration processing and is able to simulate frequency and duration judgments accurately would facilitate a better understanding of how frequency and duration are processed in the human mind. As already mentioned in the Introduction, such a model already exists in animal research (see [16]) and could be extended to human judgments of frequency and duration [56]. In such a pacemaker–accumulator model, the accumulator is incremented either in discrete steps (frequency of occurrence) or in a continuous way (duration). One could conceive of the pacemaker (the clock mechanism in such a model) as discretizing the continuous magnitude input of duration. The effect of attention might be included in the model by producing more increments or “cups” (cf. [56]), by accelerating the pacemaker, or by increasing the “filling” of each cup. Both ways would lead to higher accumulated magnitudes, yielding higher estimates by comparing the accumulated magnitudes with the respective reference memory. Such an extended accumulator model might be implemented by an associative learning mechanism that relies on a simple neural network, like the PASS (Probability ASSociator) model [82,83], that defines events or objects by their features. Using neural network models as an extension and implementation of the already existing accumulator models to simulate and predict judgments of frequency and duration could be a promising way to understand a basic cognitive process that may play an important role in many of our daily activities.

## Supporting Information

S1 Data File SPSS Data of the current study structured following the guidelines of HLM7 and the general principles of multilevel regression analysis. (XLSX)

S1 Table Stimuli (Names). (DOCX)

S2 Table Means of frequency and duration judgments for names, low-emotion pictures and high-emotion pictures under low and high physical effort. (DOCX)
