# Supplementary material for: The Impact of Attention on Judgments of Frequency and Duration
Source: PLoS One. 2015 May 22;10(5):e0126974. doi: 10.1371/journal.pone.0126974 (PMC4441377; doi:10.1371/journal.pone.0126974)
Supplement: S1 Table — (DOCX) [file pone.0126974.s002.docx]

Table S1

*Stimuli (Names)*

| Male first names | Female first names |
| --- | --- |
| Andreas | Anna |
| Christoph | Claudia |
| Daniel | Ella |
| Fabian | Karin |
| Felix | Katja |
| Florian | Klara |
| Julian | Laura |
| Lukas | Lea |
| Marcel | Lena |
| Martin | Lisa |
| Michael | Lydia |
| Moritz | Maria |
| Peter | Nadine |
| Philipp | Nina |
| Robert | Sandra |
| Stefan | Sarah |
| Thomas | Sonja |
| Tobias | Sophie |
