# Supplementary material for: The Impact of Attention on Judgments of Frequency and Duration
Source: PLoS One. 2015 May 22;10(5):e0126974. doi: 10.1371/journal.pone.0126974 (PMC4441377; doi:10.1371/journal.pone.0126974)
Supplement: S2 Table — (DOCX) [file pone.0126974.s003.docx]

Table S2

*Means of frequency and duration judgments for names, low-emotion pictures and high-emotion pictures under low and high physical effort*

| Judgment type | Stimulus duration | | | Names | | | | | Low-arousal Pictures | | | | | High-arousal Pictures | | | | |
| --- | --- | --- | --- | --- | --- | --- | --- | --- | --- | --- | --- | --- | --- | --- | --- | --- | --- | --- |
|  |  |  |  | Stimulus frequency | | | | | Stimulus frequency | | | | | Stimulus frequency | | | | |
|  |  |  |  | 2 | 4 | | | 8 | 2 | 4 | | | 8 | 2 | 4 | | | 8 |
|  | | | Low physical effort | | | | | | | | | | | | | | | |
| Frequency | 16 s | 3.76 (1.79) | | | | 4.44 (1.53) | 5.92 (2.12) | | 3.04 (1.14) | | 4.76 (1.74) | 6.56 (1.19) | | 2.88 (0.83) | | 4.32 (1.41) | 5.84 (1.49) | |
|  | 24 s | 3.60 (1.56) | | | | 4.56 (1.96) | 5.68 (1.68) | | 2.88 (0.83) | | 4.52 (1.30) | 6.48 (1.42) | | 2.96 (0.94) | | 4.12 (1.09) | 5.92 (1.66) | |
|  | 32 s | 3.68 (1.49) | | | | 4.56 (1.56) | 5.48 (1.87) | | 3.00 (1.00) | | 4.88 (1.42) | 7.04 (1.10) | | 3.44 (1.47) | | 4.72 (1.43) | 6.16 (1.77) | |
| Duration | 16 s | 20.9 (4.97) | | | | 23.0 (5.10) | 24.4 (5.05) | | 19.6 (3.98) | | 21.1 (4.91) | 24.8 (5.90) | | 19.3 (3.86) | | 20.5 (3.79) | 24.7 (5.19) | |
|  | 24 s | 21.6 (5.01) | | | | 22.6 (5.12) | 23.4 (5.58) | | 19.0 (3.35) | | 21.8 (4.70) | 26.1 (4.97) | | 19.8 (3.63) | | 23.2 (4.26) | 26.2 (5.48) | |
|  | 32 s | 19.9 (3.64) | | | | 23.4 (5.15) | 24.6 (5.68) | | 21.1 (5.14) | | 25.3 (5.45) | 28.2 (4.09) | | 23.0 (5.18) | | 25.2 (4.96) | 25.2 (4.79) | |
|  | | | High physical effort | | | | | | | | | | | | | | | |
| Frequency | 16 s | 3.64 (1.63) | | | | 4.92 (1.61) | 6.24 (1.56) | | 2.52 (0.71) | | 3.88 (1.09) | 6.24 (1.74) | | 2.64 (0.86) | | 3.48 (1.23) | 5.76 (1.59) | |
|  | 24 s | 3.32 (1.49) | | | | 4.48 (1.74) | 5.56 (1.56) | | 3.04 (0.98) | | 4.60 (1.35) | 6.60 (1.56) | | 2.48 (0.65) | | 4.64 (1.55) | 6.32 (1.46) | |
|  | 32 s | 4.12 (1.59) | | | | 5.16 (2.12) | 6.36 (1.60) | | 2.96 (1.12) | | 4.80 (1.29) | 6.92 (1.22) | | 2.96 (0.98) | | 4.32 (1.11) | 6.72 (1.46) | |
| Duration | 16 s | 22.2 (5.45) | | | | 24.2 (4.71) | 26.0 (4.17) | | 18.3 (3.08) | | 20.9 (4.31) | 23.0 (5.56) | | 17.0 (1.79) | | 20.2 (4.41) | 23.4 (4.46) | |
|  | 24 s | 21.3 (3.73) | | | | 22.9 (4.40) | 24.8 (5.22) | | 18.9 (3.24) | | 22.3 (3.97) | 27.1 (4.44) | | 18.8 (3.56) | | 22.4 (4.14) | 26.3 (3.96) | |
|  | 32 s | 22.6 (5.76) | | | | 24.0 (5.14) | 26.8 (4.46) | | 23.8 (5.86) | | 25.4 (4.61) | 29.4 (3.44) | | 21.9 (5.59) | | 25.2 (5.41) | 27.3 (4.77) | |

*Note:* Standard deviations are in parentheses.
